# *In vitro* Antimicrobial Activity of Robenidine, Ethylenediaminetetraacetic Acid and Polymyxin B Nonapeptide Against Important Human and Veterinary Pathogens

**DOI:** 10.3389/fmicb.2019.00837

**Published:** 2019-04-25

**Authors:** Manouchehr Khazandi, Hongfei Pi, Wei Yee Chan, Abiodun David Ogunniyi, Jowenna Xiao Feng Sim, Henrietta Venter, Sanjay Garg, Stephen W. Page, Peter B. Hill, Adam McCluskey, Darren J. Trott

**Affiliations:** ^1^Australian Centre for Antimicrobial Resistance Ecology, School of Animal and Veterinary Sciences, The University of Adelaide, Roseworthy, SA, Australia; ^2^School of Pharmacy and Medical Sciences, University of South Australia, Adelaide, SA, Australia; ^3^Neoculi Pty Ltd., Burwood, VIC, Australia; ^4^Chemistry, School of Environmental and Life Sciences, The University of Newcastle, Callaghan, NSW, Australia

**Keywords:** robenidine, combination, antimicrobial, canine otitis externa, EDTA

## Abstract

The emergence and global spread of antimicrobial resistance among bacterial pathogens demand alternative strategies to treat life-threatening infections. Combination drugs and repurposing of old compounds with known safety profiles that are not currently used in human medicine can address the problem of multidrug-resistant infections and promote antimicrobial stewardship in veterinary medicine. In this study, the antimicrobial activity of robenidine alone or in combination with ethylenediaminetetraacetic acid (EDTA) or polymyxin B nonapeptide (PMBN) against Gram-negative bacterial pathogens, including those associated with canine otitis externa and human skin and soft tissue infection, was evaluated *in vitro* using microdilution susceptibility testing and the checkerboard method. Fractional inhibitory concentration indices (FICIs) and dose reduction indices (DRI) of the combinations against tested isolates were determined. Robenidine alone was bactericidal against *Acinetobacter baumannii* [minimum inhibitory concentrations (MIC) mode = 8 μg/ml] and *Acinetobacter calcoaceticus* (MIC mode = 2 μg/ml). Against *Acinetobacter* spp., an additivity/indifference of the combination of robenidine/EDTA (0.53 > FICIs > 1.06) and a synergistic effect of the combination of robenidine/PMBN (0.5 < FICI) were obtained. DRIs of robenidine were significantly increased in the presence of both EDTA and PMBN from 2- to 2048-fold. Robenidine exhibited antimicrobial activity against *Escherichia coli, Klebsiella pneumoniae*, and *Pseudomonas aeruginosa*, in the presence of sub-inhibitory concentrations of either EDTA or PMBN. Robenidine also demonstrated potent antibacterial activity against multidrug-resistant Gram-positive pathogens and all Gram-negative pathogens isolated from cases of canine otitis externa in the presence of EDTA. Robenidine did not demonstrate antibiofilm activity against Gram-positive and Gram-negative bacteria. EDTA facilitated biofilm biomass degradation for both Gram-positives and Gram-negatives. The addition of robenidine to EDTA was not associated with any change in the effect on biofilm biomass degradation. The combination of robenidine with EDTA or PMBN has potential for further exploration and pharmaceutical development, such as incorporation into topical and otic formulations for animal and human use.

## Introduction

The widespread occurrence of multidrug-resistant (MDR) pathogens is problematic in both human and animal medicine (Morehead and Scarbrough, 2018). In particular, ESKAPE pathogens (*Enterococcus* spp., *Staphylococcus aureus, Klebsiella pneumoniae, Acinetobacter baumannii, Pseudomonas aeruginosa*, and *Enterobacter* spp.) deserve global attention due to the development of MDR (Santajit and Indrawattana, 2016) and increased mortality among patients (Pendleton et al., 2013). The other worrisome factor is the potential of bacteria to form biofilms that are extremely resistant to antimicrobials (Chambers and Deleo, 2009). In the past, resistance could be combated by the development of new drugs active against antimicrobial-resistant bacteria. However, the pharmaceutical industry has reduced its research efforts for the discovery and development of novel antibacterial drugs (Theuretzbacher et al., 2018). Adding to this global issue, the only novel antimicrobial classes that have been introduced in the last 20 years are the lipopeptides (daptomycin), oxazolidinones (linezolid and tedizolid) and the lipoglycopeptides (dalbavancin, oritavancin, and telavancin), which predominantly have a Gram-positive spectrum of activity (Wilcox, 2005; Saravolatz et al., 2009; Zhanel et al., 2012). The lack of novel antimicrobial development has resulted in attempts to safeguard critically important antimicrobials (antimicrobial stewardship) and a search for alternatives to treat MDR infections, including those in animals (Chan et al., 2018a; Hickey et al., 2018).

The use of critically important antimicrobials (WHO, 2017) for veterinary applications may also contribute to the development of antimicrobial resistance. For example, MDR strains of *P. aeruginosa* (MDRPA) and methicillin-resistant strains of coagulase-positive *Staphylococcus* spp. and coagulase-negative *Staphylococcus* spp., are now widespread in veterinary medicine, particularly as a cause of infections such as canine otitis, dermatitis and bovine mastitis (Beck et al., 2012; Abraham et al., 2017; Heward et al., 2018; Khazandi et al., 2018). Otitis externa is one of the most common infectious diseases in dogs, and it can be caused by both Gram-positive and Gram-negative organisms, as well as fungi. It is typically treated by topical administration of antimicrobials, such as aminoglycosides and fluoroquinolones that are critically important for human medicine (Paterson, 2016). Otitis externa treatment failures are often due to the development of antimicrobial resistance in key target pathogens, for example methicillin-resistant *Staphylococcus pseudintermedius* (MRSP) and MDRPA (Martin Barrasa et al., 2000; Heward et al., 2018). Development of antimicrobial resistance in these companion animal pathogens is a potential public health concern with documented transmission of MDRPA and MRSP occurring between humans and dogs within households (Lozano et al., 2017; Fernandes et al., 2018).

One approach that promotes antimicrobial stewardship and minimizes the likelihood of cross-resistance development and transmission between different host species is the repurposing of existing drugs for new applications. For example, monensin and narasin (polyether ionophores used as anticoccidials in animals, but not in humans), and closantel (a salicylanilide anthelmintic) have both been shown to be active against MRSP and methicillin-resistant *S. aureus* (MRSA) (Rajamuthiah et al., 2015; Chan et al., 2018a,b; Hickey et al., 2018). Robenidine is licensed as an anticoccidial agent and has been used safely worldwide since the early 1970s for control of coccidiosis in poultry and rabbits (Kantor et al., 1970; Bampidis et al., 2019). Recently, our laboratory reported that robenidine had antimicrobial activity against MRSA, vancomycin-resistant enterococci and *Streptococcus pneumoniae*, but no activity against Gram-negative bacteria unless robenidine was tested in combination with sub-inhibitory concentrations of polymyxin B nonapeptide (PMBN) (Abraham et al., 2016). The fact that robenidine only displays activity against Gram-negative organisms in the presence of PMBN is a good indication that robenidine acts on the cytoplasmic membrane of Gram-negative organisms, but is unable to breach the permeability barrier of the outer membrane (OM) (Arzanlou et al., 2017) in the absence of a membrane permeabilizer.

The spectrum of activity of antimicrobial agents can be extended by combining them with adjuvants. Two such agents, ethylenediaminetetraacetic acid (EDTA) and polymyxin B nonapeptide (PMBN), were selected for further investigation in this study. EDTA is a prescription medicine in humans given intravenously or intramuscularly for the treatment of lead poisoning (Selander, 1969), and is a component of many topically applied ointments, eye drops and ear cleaners (Guardabassi et al., 2010). EDTA is a bacteriostatic compound that permeabilizes the outer membrane of Gram-negative bacteria by chelating Ca^2+^ and Mg^2+^ cations (Vaara, 1992). In addition, EDTA has demonstrated antibiofilm activities against existing biofilms as well as preventing biofilm formation (Finnegan and Percival, 2015). PMBN derived from polymyxin B, whilst lacking antibacterial activity (except against *Pseudomonas* spp.), is able to render Gram-negative bacteria more susceptible to antimicrobials by increasing their outer membrane permeability without affecting bacterial cell viability (Schneider et al., 2017). It has been reported that the combination of PMBN with novobiocin or erythromycin administered intraperitoneally successfully treated mice infected with Gram-negative pathogens (Ofek et al., 1994; Allam et al., 2017).

Our aims in this study were to evaluate the *in vitro* antimicrobial and antibiofilm activities of robenidine either alone or in the presence of EDTA or PMBN against Gram-negative bacteria predominantly associated with otitis externa of animals and skin infections of humans, and assess the activity of the most effective combination/s against field strains of canine otitis externa pathogens including *P. aeruginosa, Proteus mirabilis, S. pseudintermedius* and beta-haemolytic streptococci. We hypothesized that either EDTA or PMBN would increase the antimicrobial activity of robenidine against Gram-negative bacteria through outer membrane permeabilization.

## Materials and Methods

### Antimicrobial Agents

Analytical grade robenidine was provided by Neoculi Pty Ltd., Burwood, VIC, Australia. The compound was stored in a sealed container in the dark at 4°C at the Infectious Diseases Laboratory, Roseworthy campus, The University of Adelaide. Polymyxin B nonapeptide (PMBN), ampicillin, apramycin, enrofloxacin, and gentamicin were purchased from Sigma-Aldrich (Australia). Stock solutions (25.6 mg/ml of PMBN in DMSO, 12.8 mg/ml of ampicillin in PBS, 12.8 mg/ml of apramycin in DMSO, 3.2 mg/ml of enrofloxacin in ½ volume of water to which was added NaOH dropwise to facilitate dissolution and 12.8 mg/ml of gentamicin in Milli-Q water) were prepared and stored in 1 ml aliquots at -80°C. They were defrosted immediately prior to use. EDTA (disodium salt) was purchased from Chem-Supply Pty Ltd., South Australia and was dissolved in Milli-Q water to 200 mM.

### Bacterial Strains

*Escherichia coli* ATCC 25922, *E. coli* ATCC 11229, *P. aeruginosa* ATCC 27853, *P*. *aeruginosa* PA01, *Pseudomonas putida* ATCC 17428, *P. mirabilis* ATCC 43071, *K. pneumoniae* ATCC 13883, *A. baumannii* ATCC 19606, and *A. baumannii* ATCC 12457 were used for preliminary susceptibility testing and combination experiments. *S. aureus* ATCC 29213 and *S. pneumoniae* ATCC 49619 were used as internal quality controls. A variety of bacterial organisms from both human and canine infections were investigated in this study (*n* = 119 isolates in total). Twenty-eight clinical *Acinetobacter* spp. isolates were obtained from cases of human skin and soft tissue infections, including 18 *Acinetobacter baumannii* and 10 *A. calcoaceticus*, kindly provided by Ms Jan Bell (Institute of Medical and Veterinary Science, South Australia). It is notable that *A. baumannii* ST2 producing OXA-23 have been reported in both humans and animals, representing a possible zoonotic lineage (van der Kolk et al., 2019). Ninety-one clinical isolates were obtained from cases of canine otitis externa, including seven methicillin-susceptible *S. pseudintermedius* (MSSP), 13 multidrug- and methicillin-resistant *S. pseudintermedius* (MRSP) (Saputra et al., 2017), 20 beta-haemolytic *Streptococcus* spp., 30 *P. aeruginosa* (10 of them resistant to gentamicin and 21 *P. mirabilis* isolates). These isolates were obtained from the bacterial collection of the national survey of antimicrobial resistance in animals conducted in Australia. Swab samples from dogs with signs of otitis externa were collected by veterinarians and submitted to government, private or university diagnostic laboratories throughout Australia. After routine bacterial identification and the removal of confidential information, the participating veterinary diagnostic laboratories submitted the bacteria and their clinical information to PC2 Laboratories, Australian Centre for Antimicrobial Resistance Ecology, School of Animal and Veterinary Sciences, University of Adelaide, Roseworthy Campus, Roseworthy, SA, Australia, for further study. Thus, animal ethics approval was not required in this study. These organisms were identified to species level using biochemical testing and MALDI-TOF mass spectrometry (Bruker, Preston, VIC, Australia).

### Antimicrobial Susceptibility Testing

Minimum inhibitory concentrations (MIC) were determined for robenidine, EDTA and PMBN in round bottom 96-well microtiter trays (Thermo Fisher Scientific, Australia), using the modified broth micro-dilution method recommended by the Clinical and Laboratory Standards Institute (CLSI, 2015). Testing concentrations were as follows: robenidine- 256–0.25 μg/ml; EDTA- 3800–45 μg/ml; PMBN- 32–0.06 μg/ml. Luria Bertani (LB) broth (Oxoid, Australia) was applied for MIC testing in lieu of cation-adjusted Mueller–Hinton broth as robenidine has been previously shown to chelate calcium ions. Furthermore, a twofold serial dilution of robenidine was performed in 100% DMSO, with 1 μl dispensed to each well due to the hydrophobicity of the compound (Abraham et al., 2016). The MIC for ampicillin or gentamicin against each isolate was determined for each test to serve as an internal quality control. The MICs of isolates were determined by visual reading and using an EnSpire Multimode Plate Reader 2300 at*A*_600_nm__. MIC_50_, MIC_90_, and MIC range for robenidine and EDTA were calculated against clinical isolates of *P. aeruginosa* and *P. mirabilis, S. pseudintermedius* and β-haemolytic *Streptococcus* spp., MIC range and MIC mode were calculated for *A. baumannii* and *A. calcoaceticus*.

### Minimum Bactericidal Concentration (MBC) Determination

The MBC of robenidine alone or in combination with EDTA or PMBN against Gram-positive and Gram-negative bacteria was determined. Briefly, 10 μl aliquots from each duplicate well from the MIC assays (starting from the MIC for each compound) were inoculated onto a sheep blood agar (SBA) plate and incubated at 37°C. Plates were examined at 24 separate intervals for a period of 2 days, the MBC was recorded as the lowest concentration of each test compound at which a 99.95% colony count reduction was observed on the plate (CLSI, 1999).

### Synergy Testing by Checkerboard Microdilution, Isobolograms and Dose Reduction Analysis

To assess the potential activity of robenidine, MICs against a range of Gram-negative ATCC strains as well as clinical isolates of canine otitis externa pathogens were performed in the presence or absence of 23.2–7,500 μg/ml (0.06–20 mM) EDTA and 0.25–128 μg/ml PMBN in a slightly modified standard checkerboard assay as described previously (Hwang et al., 2012). Briefly, antimicrobial stock solutions for robenidine and PMBN were prepared at a concentration of 12.8 mg/ml in DMSO. The antimicrobial stock solution for EDTA was prepared at a concentration of 200 mM in Milli-Q water. Then, a twofold serial dilution of each antimicrobial stock solution was prepared in its appropriate solvent (e.g., DMSO for robenidine and Milli-Q water for EDTA) from wells 12 to 3 (from 12.8 to 0.25 mg/ml for robenidine and PMBN; and 100 to 0.06 mM for EDTA). A 1 μl aliquot of the first compound from each combination was dispensed along the abscissa (from row A to G) of the 96-well microplate, while the second compound was dispensed along the ordinate (from column 12 to column 3) using an electronic multichannel pipette followed by 89 μl of LB broth. Each well of the plate was inoculated with an aliquot of 10 μl bacterial suspension at a concentration of 1-5 × 10^6^ colony forming units (CFU) per ml. Subsequently, the plate was incubated at 37°C for 24 h. The fractional inhibitory concentration index (FICI) described the results of the combinations, and was calculated utilizing the following formula:

$$\begin{array}{c} FICI of combination = FICA + FICB \end{array}$$

FIC A is the MIC of robenidine in the combination/MIC of robenidine alone, FIC B is the MIC of the adjuvant (EDTA or PMBN) in the combination/MIC of the adjuvant alone. The results indicate synergism when the corresponding FICI ≤ 0.5, additivity when 0.5 < FICI ≤ 1, indifference when 1 < FICI ≤ 4 and antagonism when the FICI > 4. In this study, the FIC for robenidine and PMBN against Gram-negative bacteria in the combination was calculated to be zero (e.g., 1 ÷ >256 = 0) when robenidine or PMBN did not show any antibacterial activity alone against Gram-negative bacteria at the highest concentration tested (e.g., 256 μg/ml), but antimicrobial activity was observed when the compounds were tested in combination.

The results of the checkerboard experiments are illustrated by isobolograms, as follows: The MIC of drug A is marked on the *x*-axis of an isobologram and the MIC of drug B on the *y*-axis, with the line connecting the two marks representing the indifferent line (no interaction) (Tallarida, 2006). The MIC values of the combination located below the indifference line indicate additive (1 ≥ FICI > 0.5) or synergistic (FICI ≤ 0.5) interactions. Values that are found above the indifferent line indicate indifferent (1 < FICI ≤ 4) or antagonistic (FICI > 4) interactions (Hwang et al., 2012).

The dose reduction index (DRI) shows the difference between the effective doses in combination in comparison to its individual dose. DRI was calculated as follows:

$$\begin{array}{c} DRI = MIC of drug alone/MIC of drug in combination \end{array}$$

Robenidine and PMBN did not show any antimicrobial activity against the majority of Gram-negative bacteria tested, the highest concentration of each compound tested against each isolate was included in the DRI equation as its MIC [e.g., the MIC of robenidine alone against *E. coli* was >256 (μg/ml) and its MIC in combination with EDTA was 1 (μg/ml); DRI = 256/1].

Dose reduction indices is very important clinically when the dose reduction is associated with a toxicity reduction without changing efficacy (Eid et al., 2012). Commonly, a DRI higher than 1 is considered beneficial.

### Time-Dependent Killing Assays

Time kill assays were performed (in duplicate) for the robenidine ± EDTA assays as described previously (CLSI, 1999) with slight modifications. Briefly, colonies of each bacterium (*P. aeruginosa* ATCC 27853, *P*. *aeruginosa* PA01, a clinical isolate of *P*. *aeruginosa* from canine otitis externa, *A. baumannii* ATCC 19606, human clinical isolates of *A*. *baumannii* B10 and *A*. *baumannii* B11) from overnight SBA plates were separately emulsified in normal sterile saline and adjusted to *A*_600_nm__ = 0.10 (equivalent to approximately 5 × 10^7^ CFU/ml). Subsequently, the bacterial suspensions were further diluted 1:10 in sterile saline. The robenidine or EDTA were serially diluted in 100% DMSO or Milli-Q water at 100× the final desired concentration and a 100 μl aliquot of appropriate concentrations added to each 10 ml preparation. Robenidine or EDTA solution was prepared in 10 ml volumes at MIC and 2× MIC concentration in LB broth. After adding inoculum dose to each tube, duplicate cultures were incubated at 37°C, with samples withdrawn at 0, 0.5, 1, 2, 4, and 24 h, serially diluted tenfold and plated on SBA overnight at 37°C for bacterial enumeration. According to CLSI, an antimicrobial agent is considered bactericidal if it causes a ≥3 × log_10_ (99.95%) reduction in CFU/ml after 18–24 h of incubation, and the combination is considered synergistic when it causes a ≥2 × log_10_ reduction in CFU/ml (Tängdén et al., 2014).

### Antibiofilm Susceptibility Testing

The minimum biofilm eradication concentration (MBEC) was determined for robenidine and EDTA using the MBEC^TM^ High-throughput assay system (MBEC^TM^ BioProducts, Innovotech, Canada) consisting of a lid with 96 pegs and a 96-well microtiter plate as previously described (Ceri et al., 2001; Harrison et al., 2010). Briefly, biofilms of *P. aeruginosa* PA01, two clinical isolates of *P. aeruginosa* isolates and two clinical isolates of *S. pseudintermedius* were formed by inoculating 150 μl of 10^7^ CFU/ml of each bacterial suspension in the MBEC^TM^ device. The inoculated device was aerobically incubated on an orbital shaker at 37°C (OM11, Ratek Instruments Pty Ltd., Australia) for 24 h to produce equivalent (Uniform) biofilms on all pegs. Biofilms of *P. aeruginosa* and *S. pseudintermedius* were exposed to challenge plates containing a serial concentration of robenidine (from 0.125 to 128 μg/ml) or EDTA (1–32 mM) and incubated at 37°C for 24 h. Following antimicrobial challenge, the biofilms were rinsed twice with phosphate buffered saline (pH = 7) and disrupted via sonication (Soniclean, Model 160TD, Australia) for 10 min into the recovery medium. Viable cell counts were determined for recovered cells (colony-forming units per peg) after preparing a serial dilution and plating 10 μl in duplicates of each dilution onto plate count agar. Viable counts were then expressed as a percentage of the mean CFU of growth controls. MBEC was defined as the lowest concentration of antimicrobial agent that eradicates the biofilms recovered from the antimicrobial challenge.

### Checkerboard Microdilution Assay for Antibiofilm Activity of Robenidine

A slightly modified standard checkerboard assay was used to determine the activity of robenidine in the presence or absence of 37.2–12,000 μg/ml (1–32 mM) EDTA as described previously (Hwang et al., 2012). Briefly, the MBEC^TM^ High-throughput assay system (MBEC^TM^ BioProducts, Innovotech, Canada) was used for the preparation of Gram-positive and Gram-negative biofilm producing bacteria as described above for antibiofilm susceptibility testing. The antimicrobial stock solution for EDTA was prepared at a concentration of 128 mM in Milli-Q water and robenidine was prepared at 12.8 mg/ml in DMSO. Then, a twofold serial dilution of each antimicrobial stock solution was prepared in its appropriate solvent from wells 12 to 3 (from 12.8 to 0.25 mg/ml for robenidine and 128 to 1 mM for EDTA). A 2 μl aliquot of robenidine compound from each concentration was dispensed along the abscissa (from row A to H) of the 96-well microplate, while 100 μl of EDTA was dispensed along the ordinate (from column 12 to column 3) using an electronic multichannel pipette followed by 98 μl of LB broth. Subsequently, the plate was incubated at 37°C for 24 h.

### *In vitro* Cytotoxicity Assays

A panel of adherent mammalian cell lines, HaCat (human immortalized keratinocytes), HEK 293 (human embryonic kidney) and MDCK (normal Madin Darby Canine Kidney) were assayed for *in vitro* cytotoxicity of robenidine alone or in combination with EDTA or PMBN. Cells were maintained in Dulbecco’s modified Eagle’s medium (DMEM) with 10% fetal bovine serum (FBS) and 1% PenStrep (100 U/mL Penicillin and 100 μg/mL Streptomycin) at 37°C with 5% CO_2_. Cells were serially passaged at ∼80% confluence ∼ every 4 days. Assays were performed in duplicate in 96 well plates seeded with ∼25,000 cells per well. After 24 h, media was removed, washed once with medium without antimicrobials and replaced with fresh media to which robenidine in the presence or absence of EDTA and PMBN were added same concentrations used for antimicrobial susceptibility testing. Briefly, the antimicrobials were prepared by performing a twofold serial dilution at 100× of tested concentration in DMSO. Subsequently, a 1 μl aliquot of each concentration was transferred to a sterile 96-well plate containing fresh DMEM with either 10% FBS. After mixing four times, the media aliquots with different concentrations of antimicrobial were transferred to each well of the 96-well plate seeded with cells, using wells containing 1–2% DMSO only as control. To determine the effect of FBS on the cytotoxicity of each compound, DMEM with 40% FBS containing different concentrations of antimicrobial was prepared as described above. After 24 h of exposure, WST-1 reagent (Cell Proliferation Assay reagent, Roche) at a concentration of 10% was added to each well. Absorbance at *A*_450 nm_ on a Multiskan Ascent 354 Spectrophotometer (Labsystems) was measured after 1 h of incubation. The IC_50_ value was determined for each compound against each cell line via non-linear regression (three parameters) using GraphPad Prism v6 software.

## Results

### Antimicrobial Activity of Robenidine Against Gram-Negative Control Strains

Robenidine did not demonstrate any antimicrobial activity against Gram-negative control strains (*E. coli* ATCC 25922, *E. coli* ATCC 11229, *P. aeruginosa* ATCC 27853, *P. mirabilis* ATCC 43071 and *K. pneumoniae* ATCC 13883) at the highest concentrations (256 μg/ml) tested except for *A. baumannii* ATCC 19606 (32 μg/ml) and *A. baumannii* ATCC 12457 (64 μg/ml).

### Antimicrobial Activity of Robenidine Against Human Clinical *Acinetobacter* spp.

The MIC results of robenidine against *A. baumannii* and *A. calcoaceticus* isolated from human clinical cases were demonstrated at concentrations ranging from 8 to 64 μg/ml (MIC mode = 8 μg/ml) for 18 *A. baumannii* and 1–8 μg/ml (MIC mode = 2 μg/ml) for 10 *A. calcoaceticus.* The ratio of MBC/MIC values for both *Acinetobacter* spp. was either 2× or 4× their MICs.

### Combination of Robenidine With EDTA or PMBN Against Gram-Negative Control Strains

The presence of EDTA in combination with robenidine was associated with a notable increase in the potency and spectrum of activity against Gram-negative control strains. The results of MIC and DRI values for the combination of robenidine and EDTA against *E. coli* ATCC 25922, *E. coli* ATCC 11229, *P. aeruginosa* ATCC 27853, *P. mirabilis* ATCC 43071, *K. pneumoniae* ATCC 13883, *A. baumannii* ATCC 19606, and *A. baumannii* ATCC 12457 are presented in Table 1. The combination of robenidine and EDTA resulted in a synergistic interaction against the standard isolates of *E. coli* as well as against *P. aeruginosa* ATCC 27853, *P. putida* ATCC 17428, *P. aeruginosa*, and *K. pneumoniae* ATCC 13883. An additive/indifferent interaction was recorded against *P. mirabilis, A. baumannii*, and *A. calcoaceticus* control strains. DRIs of robenidine were significantly increased in the presence of EDTA from 2- to 256-fold (Table 1).

**Table 1:** The MIC (μg/ml) values for robenidine, EDTA and the combination effect of EDTA on the MIC of robenidine for Gram-negative control strains and a human clinical *A. calcoaceticus* isolate.

Isolates	MIC (μg/ml; mM concentrations in parentheses)			Combination Effect (FICI)^b^	DRI^c^
	Single drug		Combination		
	EDTA	ROB^a^	EDTA:ROB		EDTA:ROB
*E. coli* ATCC 25922	3800 (10)	>256	950 (2.5)**:**4	Synergism (0.25)	4**:**64
*E. coli* ATCC 11229	950 (2.5)	>256	228 (0.6)**:**8	Synergism (0.25)	4**:**32
*P. putida* ATCC 17428	1900 (5)	>256	950 (2.5)**:**1.25	Synergism (0.5)	2**:**256
*P. aeruginosa* PA01	1900 (5)	>256	950 (2.5)**:**1.25	Synergism (0.5)	2**:**256
*P. aeruginosa* ATCC 27853	3800 (10)	>256	1900 (5)**:**1.25	Synergism (0.5)	2**:**256
*P. mirabilis* ATCC 43071	228 (0.6)	>256	228 (0.6)**:**1.25	Additivity (1)	1**:**256
*K. pneumoniae* ATCC 13883	11400 (30)	>256	3800 (10)**:**128	Synergism (0.33)	3**:**2
*A. baumannii* ATCC 19606	380 (1)	32	190 (0.5)**:**4	Additivity (0.62)	2**:**8
*A. baumannii* ATCC 12457	190 (0.5)	64	95 (0.25)**:**2	Additivity (0.53)	2**:**32
*A. calcoaceticus*	228 (0.6)	4	228 (0.6)**:**0.125	Indifference (1.06)	1**:**32

*^*a*^ROB, robenidine; ^*b*^the results indicate synergism when the corresponding FICI ≤ 0.5; additivity when 0.5 < FICI ≤ 1, indifference when 1 < FICI ≤ 4 and antagonism when the FICI > 4; ^*c*^DRI, dose reduction index*.

The results of MIC, FICI, and DRI values for the combination of robenidine and PMBN against *E. coli* ATCC 25922, *E. coli* ATCC 11229, *P. aeruginosa* ATCC 27853, *P. mirabilis* ATCC 43071, *K. pneumoniae* ATCC 13883, *A. baumannii* ATCC 19606, and *A. baumannii* ATCC 12457 are presented in Table 2. The combination of robenidine and PMBN resulted in a synergistic interaction against all the isolates tested except *P. mirabilis* ATCC 43071 (Table 2). DRIs of robenidine were significantly increased in the presence of PMBN from 8- to 256-fold (Table 2).

**Table 2:** The MIC (μg/ml) values for robenidine, PMBN and the combination effect of PMBN on the MIC of robenidine for Gram-negative control strains and a human clinical *A. calcoaceticus*.

Isolates	Antimicrobial concentration (μg/ml)			Combination Effect (FICI)^c^	DRI^d^
	Single drug		Combination		
	PMBN^a^	ROB^b^	PMBN:ROB		EDTA:ROB
*E. coli* ATCC 25922	>32	>256	6:8	Synergism (<0.5)	5:32
*E. coli* ATCC 11229	>32	>256	6:8	Synergism (<0.5)	5:32
*P. putida* ATCC17428	4	>256	1:1	Synergism (0.25)	4:256
*P. aeruginosa* ATCC27853	2	>256	0.75:1	Synergism (0.25)	4:256
*P. mirabilis* ATCC 43071	>32	>256	NA^e^	No effect	NA
*K. pneumoniae* TCC13883	>32	>256	0.5:4	Synergism (<0.5)	64:64
*A. baumannii* ATCC19606	>32	32	1:2	Synergism (0.07)	32:16
*A. baumannii* ATCC 12457	>32	64	1:2	Synergism (0.03)	32:32
*A. calcoaceticus*	>32	4	1.5:0.5	Synergism (0.12)	21:8

*^*a*^PMBN, polymyxin B nonapeptide; ^*b*^ROB, robenidine; ^*c*^the results indicate synergism when the corresponding FICI ≤ 0.5; additivity when 0.5 < FICI ≤ 1, indifference when 1 < FICI ≤ 4 and antagonism when the FICI > 4; ^*d*^DRI, dose reduction index; ^*e*^NA, no antimicrobial activity was recorded.*

**FIGURE 1:**
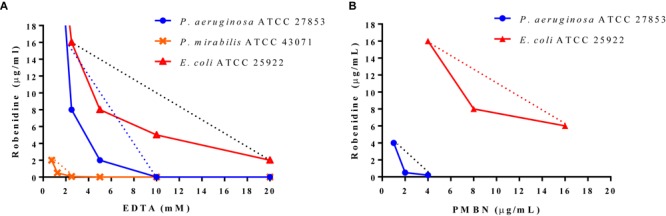
Isobologram analyses. Minimum inhibitory concentrations of **(A)** EDTA and **(B)** PMBN are plotted on *x*-axis and minimum inhibitory concentration values of robenidine on *y*-axis. The curves represent the combinations against *E. coli* ATCC 25922, *P. mirabilis* ATCC 43071 and *P. aeruginosa* ATCC 27853 including the indifference line (.......) for each isolate.

Isobologram analyses were carried out for the combination of EDTA and robenidine against *E. coli* ATCC 25922, *P. aeruginosa* ATCC 27853 and *P. mirabilis* ATCC 43071, and for the combination of PMBN and robenidine against *E. coli* ATCC 25922 and *P. aeruginosa* ATCC 27853. Dose-effect curves for drugs with different maxima and the corresponding isobole combination are presented in Figure 1. Isoboles of the combination of EDTA and robenidine against *E. coli* ATCC 25922 and *P. aeruginosa* ATCC 27853 indicated synergism. Similarly, isoboles of the combination of PMBN and robenidine against *E. coli* ATCC 25922 and *P. aeruginosa* ATCC 27853 also indicated synergism.

### Antimicrobial Activity of Robenidine Against Canine Otitis Externa Pathogens

MIC range, MIC_50_, MIC_90_ (μg/ml) values of robenidine, gentamicin, apramycin, and ampicillin against quality control strains (*S. aureus* ATCC 29213, *P. aeruginosa* ATCC 27853, *E. coli* ATCC 25922) and clinical isolates from otitis externa cases in dogs [*S. pseudintermedius* (*n* = 20), beta-haemolytic streptococci (*n* = 20), *P. mirabilis* (*n* = 21), and *P. aeruginosa* (*n* = 30)] are presented in Table 3.

**Table 3:** The MIC range, MIC_50_, MIC_90_ (μg/ml) values of robenidine, gentamicin, apramycin, and ampicillin against control strains, including *P. aeruginosa* ATCC 27853, *E. coli* ATCC 25922 *S. aureus* ATCC 29213, *S. pneumoniae* ATCC 49619 and clinical isolates from otitis externa cases in dogs, including *S. pseudintermedius* (*n* = 20), beta-haemolytic *Streptococci* (*n* = 20), *P. mirabilis* (*n* = 21), and *P. aeruginosa* (*n* = 30).

	Value	MIC (μg/ml)			
		Robenidine	Gentamicin	Apramycin	Ampicillin
*P. aeruginosa* (*n* = 30)	MIC range	>256	0.25–64	16–64	–a
	MIC_50_	>256	32	32	–
	MIC_90_	>256	64	64	–
*P. mirabilis* (*n* = 21)	MIC range	>256	4–> 128	16–64	–
	MIC_50_	>256	8	32	–
	MIC_90_	>256	32	64	–
*S. pseudintermedius* (*n* = 20)	MIC range	1–4	1–64	1–16	0.03–32
	MIC_50_	2	2	8	0.125
	MIC_90_	2	2	16	8
Beta-haemolytic streptococci (*n* = 20)	MIC range	4–16	8–16	4–128	0.06–0.5
	MIC_50_	8	8	64	0.125
	MIC_90_	8	8	128	0.25
**Quality control strains**					
*S. aureus* ATCC 29213	MIC	2	0.5	4	1
*P. aeruginosa* ATCC 27853	MIC	–	2	16	–
*E. coli* ATCC 25922	MIC	–	0.5	8	4
*S. pneumoniae* ATCC 49619	MIC	–	–	–	0.125

*^*a*^Antimicrobial activity was not tested.*

### Combination of Robenidine With EDTA Against Canine Otitis Externa Pathogens

Minimum inhibitory concentrations and DRI values for the combination of robenidine and EDTA against 30 *P. aeruginosa*, 21 *P. mirabilis*, 20 *S. pseudintermedius*, and 20 beta-haemolytic streptococci isolated from canine otitis externa cases are shown in Table 4. The clinical isolates of *P. aeruginosa* including 10 antimicrobial-resistant isolates, showed a synergistic interaction with the combination of robenidine and EDTA. An additivity interaction (95.3%) was recorded against clinical isolates of *P*. *mirabilis* in the combination of robenidine and EDTA. The DRIs of robenidine for *P. aeruginosa* and *P*. *mirabilis* isolates increased between 64- and 2048-fold, and the DRIs of EDTA increased two and fourfold. Additionally, additive and indifferent activity of the combination robenidine and EDTA was observed against clinical isolates of MRSP, MSSP and beta-haemolytic streptococci.

**Table 4:** The MIC range, MIC_50_, MIC_90_ (μg/ml) and DRI values for robenidine, EDTA alone and their combination against 30 *Pseudomonas aeruginosa*, 21 *Proteus mirabilis*, 20 *Staphylococcus pseudintermedius*, and 20 beta-haemolytic streptococci isolated from otitis externa cases in dogs.

Isolates	Value	Antimicrobial concentration (μg/ml; mM concentrations in parentheses)				Combination Effect^b^ (percentage)	DRI^c^	
		Single drug		Combination				
		EDTA	ROB^a^	EDTA	ROB		EDTA	ROB
*P. aeruginosa* [*n* = 30; MSSP (20), MRSP (10)]	MIC range	750–4500 (2–12)	>256	380–1500 (1–4)	0.125–4	Synergism (100%)	2–3	64–2048
	MIC_50_	3000 (8)	>256	1500 (4)	0.25		2	1024
	MIC_90_	3000 (8)	>256	1500 (4)	2		2	128
	MBC/MIC	BS^d^	NA^e^	750 (2)	≥2	–		
*P. mirabilis* (*n* = 21)	MIC range	190–750 (0.5–2)	>256	190–380 (0.5–1)	0.125–4	Additivity (95.3%) Synergism (4.7%)	1–2	64–2048
	MIC_50_	380 (1)	>256	380 (1)	0.125		2	2048
	MIC_90_	750 (2)	>256	380 (1)	0.5		2	512
	MBC/MIC	BS	NA	750 (2)	≥2	–		
*S. pseudintermedius* [*n* = 20; MSSP (7), MRSP (13)]	MIC range	95–380 (0.25–1)	1–4	95 (0.25)	0.25–2	Additivity (100%)	1–4	2–8
	MIC_50_	190 (0.5)	2	95 (0.25)	1		2	2
	MIC_90_	190 (0.5)	2	95 (0.25)	1		2	2
	MBC/MIC	BS	1.5	750 (2)	2	–		
Beta-haemolytic streptococci (*n* = 20)	MIC range	190–750 (0.5–2)	4–16	95–380 (0.25–1)	1–8	Additivity (100%)	1–2	2–8
	MIC_50_	380 (1)	8	190 (0.5)	2		2	4
	MIC_90_	750 (2)	8	380 (1)	4		1	2
	MBC/MIC	BS	1.75	750 (2)	2	–		

*^*a*^ROB, robenidine; ^*b*^ the results indicate synergism when the corresponding FICI ≤ 0.5; additivity when 0.5 < FICI ≤ 1, indifference when 1 < FICI ≤ 4 and antagonism when the FICI > 4; ^*c*^DRI, dose reduction index; ^d^BS, bacteriostatic compound; ^*e*^NA, no antimicrobial activity was recorded*.

Robenidine demonstrated antibacterial activity against Gram-positive bacteria, with MIC values ranging from 1 to 16 μg/ml against *S. pseudintermedius* and beta-haemolytic streptococci, respectively. The lowest level of interaction was recorded for the combination of robenidine and EDTA against *S. pseudintermedius* and beta-haemolytic streptococci. However, the dose reduction for beta-haemolytic streptococci ranged from 2- to 16-fold for robenidine and twofold for EDTA.

### Time Kill Kinetics of Drug Combinations Against *P. aeruginosa* and *A. baumannii*

**FIGURE 2:**
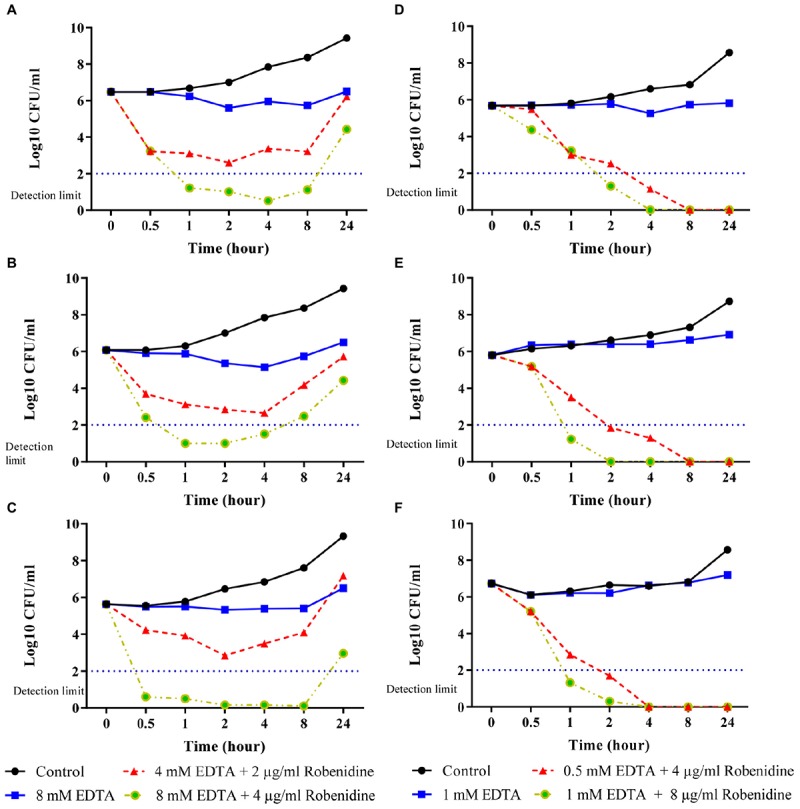
Time kill curves of EDTA and the combination of robenidine and EDTA against **(A)** *Pseudomonas aeruginosa* ATCC 27853, **(B)** *P*. *aeruginosa* PA01, **(C)** a clinical isolate of *P*. *aeruginosa* from dog **(D)** *Acinetobacter baumannii* ATCC 19606, **(E)** a clinical isolate of *A. baumannii* B10 from human and **(F)** a clinical isolate of *A*. *baumannii* B11 from human. Control represents bacteria incubated in the absence of EDTA and robenidine. Bactericidal activity of robenidine in the combination with EDTA was defined as a reduction in the numbers of viable bacteria of ≥3 log_10_ CFU/ml at any incubation time tested.

Time kill curves for robenidine in the presence of EDTA at the concentration of MIC_90_ (2 μg/ml robenidine + 1,500 μg/ml or 4 mM of EDTA) and 2× MIC_90_ (4 μg/ml robenidine + 3,000 μg/ml or 8 mM of EDTA) were obtained for *P. aeruginosa* ATCC 27853, *P. aeruginosa* PA01 and a clinical isolate of *P. aeruginosa* from a canine otitis externa case are presented in Figure 2A–C. The combination of robenidine and EDTA at MIC_90_ significantly reduced the colony count of *P. aeruginosa* isolates (about 3 log_10_) over 0.5, 1, 2, and 4 h with a synergistic effect in comparison to the control growth and EDTA alone. However, at 24 h, bacterial regrowth was observed to almost the same level as the sample treated with EDTA alone. Further reductions of the bacteria (greater than 5 log_10_ CFU/ml reduction) at 0.5 h were recorded when the EDTA concentration increased from MIC_90_ (3,000 μg/ml or 4 mM) to 2× MIC_90_ (3,000 μg/ml or 8 mM) in comparison to control and EDTA alone. A minimum of a 5 log_10_ reduction was still evident at 4 h incubation, however, after 24 h the numbers of bacteria present had increased. However, this reduction (approximately 5 log_10_ reduction) remained consistent in comparison to growth control.

Time kill curves for robenidine in the presence of EDTA at the concentration of MIC (4 μg/ml robenidine + 188 μg/ml or 0.5 mM of EDTA) and 2× MIC (8 μg/ml robenidine + 376 μg/ml or 1 mM of EDTA) for *A. baumannii* ATCC 19606, two human clinical isolates of *A*. *baumannii* (B10 and B11) from canine otitis externa are presented in Figure 2D–F. The combination of robenidine and EDTA at both MIC and 2× MIC significantly reduced the colony counts of *A. baumannii* ATCC 19606 and two clinical isolates of *A. baumannii* over 1 and 2 h with a synergistic effect in comparison to the control growth and EDTA alone. After 8 and 4 h, bacteria were eliminated for tested isolates in both MIC and 2× MIC, respectively.

### Antibiofilm Activity of Robenidine Alone and in the Presence of EDTA

**FIGURE 3:**
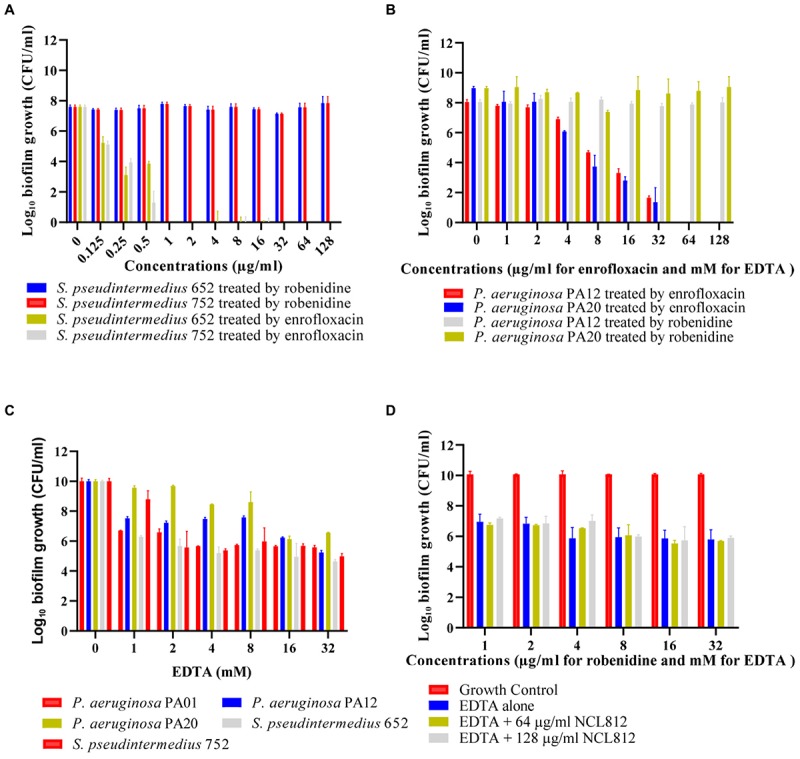
Antibiofilm activity of robenidine, EDTA, the combination of robenidine and EDTA and enrofloxacin as a control against *P*. *aeruginosa* PA01, clinical isolates of *P. aeruginosa* (PA12 and PA20) and *S. pseudintermedius* (652 and 752) from dogs. **(A)** antibiofilm activity of robenidine against *S. pseudintermedius* isolates (652 and 752), **(B)** antibiofilm activity of robenidine against *P*. *aeruginosa* PA01 and clinical isolate of *P. aeruginosa*, **(C)** antibiofilm activity of EDTA against *P*. *aeruginosa* PA01, two clinical isolates of *P. aeruginosa* (PA12 and PA20) and *S. pseudintermedius* (652 and 752), and **(D)** antibiofilm activity of the EDTA in the present of robenidine against *P*. *aeruginosa* PA01.

Preformed biofilms of *P*. *aeruginosa* PA01, two clinical isolates of *P. aeruginosa* and two clinical isolates of *S. pseudintermedius* were tested against robenidine and EDTA to determine their activities. Robenidine at concentration of up to 128 μg/ml did not show any antibiofilm activity against *P. aeruginosa* and *S. pseudintermedius* isolates in comparison to enrofloxacin as a positive control (Figure 3A,B). However, 1 mM concentration of EDTA demonstrated a significantly effect in disrupting the 24 h preformed biofilms in comparison to growth control against both Gram-positive and Gram-negative bacteria (Figure 3C). EDTA was more effective against the biofilms when the concentration of EDTA increased to 16 mM. However, the presence of robenidine in combination with EDTA was not associated with any change in the antibiofilm activity of EDTA against both Gram-positive and Gram-negative bacteria. The results of the antibiofilm activity of the EDTA in the present of robenidine against *P*. *aeruginosa* PA01 are shown in Figure 3D.

### Robenidine Cytotoxicity to Mammalian Cell Lines

The cytotoxicity profile of robenidine in the presence and absence of EDTA and PMBN was evaluated in a panel of different cultured mammalian cells using the WST-1 Cell Proliferation Assay reagent (Roche). The results of the *in vitro* cytotoxicity measurements show IC_50_ values of 12 μg/ml for robenidine, IC_50_ values of 3.4 mM for EDTA, while PMBN gave IC_50_ values of >32 μg/ml against all the cell lines tested (Table 5). We found that the *in vitro* cytotoxicity measurements show IC_50_ values of 12 μg/ml for robenidine in the presence of either 3.4 mM for EDTA or 32 μg/ml for PMBN (Table 5). Real-time cell viability measurements using HaCaT and HEK 239 cell lines also confirmed no measurable effect on cell viability for robenidine at either 12 μg/ml up to 24 h post-treatment alone or in the presence of either 3.4 mM for EDTA or 32 μg/ml for PMBN. Real-time cell viability showed that the combination of 8 μg/ml robenidine with 4 Mm EDTA was not toxic during the first 12 h of assays. Importantly, the toxicity of robenidine alone or in the presence of either EDTA or PMBN was significantly reduced from 12 μg/ml to higher than 32 μg/ml for all tested cell lines when the amount of FBS was increased from 10 to 40% (Table 5).

**Table 5:** IC_50_ data for robenidine, EDTA, PMBN and robenidine in the combination with either EDTA or PMBN against the HaCaT, HEK 293, and MDCK cell lines in the presence of 10 or 40% FBS in DMEM.

	IC_50_ values (μg/ml for robenidine and PMBN; mM for EDTA)									
Agent	ROB^a^		EDTA		PMBN		ROB:EDTA		ROB:PMBN	
FBS	10%^b^	40%^c^	10%	40%	10%	40%	10%	40%	10%	40%
HaCaT	14	>32	3.8	3.8	>32	>32	12:3	>32:3	12:>32	>32:>32
HEK 293	12	>32	3.4	3.4	>32	>32	12:3	>32:3	12:>32	>32:>32
MDCK	12	>32	3.4	3.4	>32	>32	12:3	>32:3	12:>32	>32:>32

*^*a*^ROB, robenidine, ^*b*^DMEM with 10% FBS used for cytotoxicity, ^*c*^DMEM with 40% FBS used for cytotoxicity*.

## Discussion

Bacterial pathogens have developed numerous resistance strategies against antimicrobial agents used in both humans and animals. A major challenge in successful treatment of bacterial infections is the emergence and rapid global spread of multidrug-resistant clones that are refractory to current antimicrobial therapy. To address this problem, we have examined and repurposed robenidine as a new class of antibacterial agent. To evaluate the potential of robenidine as an antibacterial agent, we previously assessed its potency, metabolic stability, pharmacokinetic and safety profiles, in a mouse PK study and a series of *in vitro* efficacy and cell toxicity studies (Abraham et al., 2016; Ogunniyi et al., 2017). We identified that robenidine had a predominantly Gram-positive spectrum of activity, and that the site of action was likely to be the cytoplasmic membrane (Ogunniyi et al., 2017) hence this compound should potentially have an antimicrobial effect on Gram-negative organisms. The Gram-positive selective activity of robenidine is most likely to be a result of the inability of this compound to traverse the outer membrane of Gram-negative organisms (Arzanlou et al., 2017). In the present study, we extended our analyses by assessing *in vitro* efficacy against a range of clinical human and animal Gram-negative bacterial isolates in the presence or absence of sub-inhibitory concentrations of EDTA and PMBN.

We found that robenidine showed antimicrobial activity against *Acinetobacter* spp. even in the absence of OM permeabilisation, and its MICs were reduced 8- to 32-fold in the presence of EDTA and PMBN. This result is quite surprising as the permeability of the OM of *A. baumannii* is estimated to be only 1–8% that of *E. coli* as *A. baumannii* lacks the general, non-specific trimeric porins found in *E. coli* (Nikaido, 2003; Zgurskaya et al., 2015). The general architecture of the OM between *A. baumannii* and other Gram-negative bacteria is the same, however, lipid A in *A. baumannii* is acylated with C12 and C14 fatty acids, compared with C10 and C12 fatty acids in *E. coli* (Zgurskaya et al., 2015). As a result, the hydrophobic core of *A. baumannii* is expected to be thicker and lipid A should occupy a larger area per lipid. These features are likely to make the OM of *A. baumannii* more hydrophobic and could be responsible for the susceptibility of this organism to amphiphilic antimicrobials such as novobiocin and tetracycline (Krishnamoorthy et al., 2017). Similarly, robenidine is an amphiphilic molecule and the same differences in the OM of *A. baumannii* could increase its susceptibility to robenidine.

Given these encouraging results for antibacterial activity against *Acinetobacter* spp., the safety and efficacy of robenidine could be further explored in animal models of *Acinetobacter* infection (Paluchowska et al., 2017; Gorla et al., 2018) prior to further clinical development. In addition, using an appropriate formulation can improve the potency and safety of robenidine as a novel treatment for infections caused by *A. baumannii* (Paluchowska et al., 2017; Gorla et al., 2018) and *A. baumannii*-*calcoaceticus* complex (Clark et al., 2016; Ozvatan et al., 2016), which are reported to be emerging pathogens worldwide (Gales et al., 2001). Our results show that EDTA would be a suitable adjuvant for topical delivery but not systemic use due to the high concentrations of Ca^2+^ and Mg^2+^ in blood, while PMBN does not have this limitation and could be included as a possible adjuvant in both topical and systemic *Acinetobacter* infection models.

Robenidine in the presence of sub-inhibitory concentrations of EDTA or PMBN also displayed improved antibacterial activity against a variety of ESKAPE isolates (*S. aureus, E. coli, K. pneumoniae, A. baumannii*, and *P. aeruginosa*). In the case of PMBN, it resulted in a 4- to 256-fold increase in the susceptibility of tested Gram-negative ATCC strains of ESKAPE pathogens in combination with robenidine, inhibiting growth at robenidine concentrations as low as 0.125 μg/ml, whilst the MIC of PMBN was reduced 4- to 64-fold when used in combination for *P. aeruginosa*. Our cytotoxicity results showed that robenidine (IC_50_ = 12 μg/ml) was not toxic at the MIC_90_ (0.5–4 μg/ml) of the tested pathogens, with IC_50_/MIC ratio ranging from sixfold (Gram-negative pathogens) to threefold (Gram-positive pathogens) in the presence of EDTA. The MIC (0.5–8 μg/ml) obtained for robenidine in the presence of PMBN against Gram-negative pathogens was not toxic against all tested cell lines, with IC_50_/MIC ratio ranging from approximately 2- to 24-fold. In this study, we found that toxicity of robenidine was significantly reduced in the presence of serum, possibly due to the interaction between robenidine and serum. This serum impact was observed on the MIC values of robenidine with 10% serum (fourfold increase) and 50% serum (no antimicrobial activity), which was reported in a previous study (Abraham et al., 2016). This suggests the probable high level of serum protein binding with robenidine may significantly reduce its toxicity and robenidine would be likely to be safe when applied as a topical or otic treatment. However, testing in animal models would be required to confirm efficacy and safety. In addition, the use of EDTA in topical treatments containing robenidine also is expected to be safe. EDTA-tromethamine solution consisting of 250 mM EDTA and 50 mM tromethamine has previously been used for the treatment of otitis externa, dermatitis and cystitis without any toxicity or other side effects observed (Farca et al., 1997). Given the substantial reduction in MICs and toxicity of robenidine in the presence of either EDTA or PMBN, the *in vivo* activity of these combinations for topical and systemic treatment of ESKAPE pathogen infections could be evaluated in mouse models of infection.

We found that robenidine has no activity against biofilms formed by Gram-positive or Gram-negative bacteria. However, in this study EDTA demonstrated antibiofilm activity against both Gram-positive and Gram-negative species at a concentration of 1 mM that is in agreement with previous studies (Al-Bakri et al., 2009; Finnegan and Percival, 2015). Our results demonstrate that the presence of robenidine does not affect the antibiofilm activity of EDTA. Many pathogens are able to form biofilms making them less susceptible to various classes of antimicrobials (Chambers and Deleo, 2009). There is an urgent need for antimicrobials that can either kill planktonic cells or eradicate biofilms. Together, our results show that the combination of EDTA and robenidine is a suitable antimicrobial combination with activity against both Gram-positive and Gram-negative species and their biofilm formation.

Commercially available otic products typically contain antifungal, antibiotic and anti-inflammatory agents, such as Surolan^®^ (polymyxin B-miconazole-prednisolone), Aurizon^®^ (marbofloxacin-clotrimazole-dexamethasone) and Otomax^®^ (gentamicin-clotrimazole-betamethasone) (Rougier et al., 2005; Rigaut et al., 2011). These otic products share antimicrobial agents used in human medicine, increasing the likelihood of cross-resistance development and transmission between different host species. In addition, the response to these otic products varies due to the emergence of antimicrobial resistance in canine otic pathogens. Polymyxin B resistance was reported in 100% of *S. pseudintermedius* and *Proteus* spp. and 7% of *P. aeruginosa* from cases of canine otitis externa in Australia (Bugden, 2013) and between 9.6 and 27% of canine otitis/pyoderma isolates were resistant to marbofloxacin (Rubin et al., 2008; Arais et al., 2016). Resistance to gentamicin was found in 43.3% *P. aeruginosa* otitis isolates (Mekic et al., 2011). It is notable that there is no study that demonstrates an otic product with 100% cure rate. For instance, cure rates of 58.3% for Aurizon^®^ and 41.2% for Surolan^®^ were observed in one study (Rougier et al., 2005). We found that the new combination of robenidine and EDTA has potential for development as a topical treatment of canine otitis externa with mixed bacterial infections. In our study, EDTA acted as an adjuvant that potentiates the activity of robenidine against Gram-negative bacteria with additional inhibitory activity against biofilm-forming bacteria. The use of an antimicrobial and an antimicrobial adjuvant as a two-drug combination antimicrobial therapy such as robenidine and EDTA has the benefit of reducing the onset of resistance development compared to monotherapy (Worthington and Melander, 2013). Recently, we reported that EDTA has anti-fungal activity against *Malassezia pachydermatis* isolated from canine otitis externa (Chan et al., 2018c) which is an advantage to the use of combination therapy of robenidine and EDTA for canine otitis externa. This combination is an approach to promote antimicrobial stewardship by eliminating the likelihood of cross-resistance development and transmission of resistance determinants of public health significance between dogs and humans.

In our study, robenidine demonstrated noteworthy activity against thirteen multidrug- and methicillin-resistant *S. pseudintermedius* and 20 β-haemolytic streptococci isolates from clinical cases of canine otitis externa. This is in agreement with our previous study that reported robenidine was effective against clinical MRSA and *S. pneumoniae* strains at concentrations ranging from 1–2 μg/ml and 2–8 μg/ml, respectively (Abraham et al., 2016; Ogunniyi et al., 2017). The finding that robenidine in the presence of EDTA demonstrated antibacterial activity against the Gram-negative canine otitis externa pathogens, *P. aeruginosa* and *P. mirabilis* is in agreement with our previous findings for robenidine tested against two strains each of *E. coli* and *P. aeruginosa* in the presence of PMBN (Abraham et al., 2016). However, our results showed that low concentrations of robenidine in combination with PMBN or EDTA improved potency and spectrum of activity, specifically targeting Gram-negative pathogens. These results suggest that in addition to having excellent activity against Gram-positive organisms, robenidine in combination with EDTA or PMBN has potential as a broad-spectrum topical treatment, particularly against pathogens that have become resistant to multiple classes of currently registered antimicrobial agents.

## Conclusion

The results of our study demonstrate that robenidine is not suitable as a sole antimicrobial agent for the treatment of Gram-negative pathogen infections due to the lack of activity against the majority of Gram-negative isolates except for *A. baumannii* and *A. calcoaceticus*. However, we demonstrated *in vitro* efficacy against all selected Gram-negative organisms when robenidine was tested in combination with EDTA or PMBN, including against multidrug-resistant strains. Therefore, robenidine may be an appropriate candidate as a component of a combination preparation for the treatment of otitis externa in dogs. This study provides proof of concept of drug repurposing in the field of veterinary otology and would represent a good example of antimicrobial stewardship when the compound is ultimately developed and used clinically in dogs. Finally, the additive and synergistic effects of robenidine in combination with EDTA or PMBN provide an important and novel development pathways for treatment of additional antimicrobial-resistant Gram-negative pathogens in animals and humans.

## Author Contributions

MK contributed to the study design, MIC and combination testing, kill time, biofilm assay, analyzed results, and wrote the preliminary manuscript. HP contributed towards kill time assay, MIC testing, biofilm assay, and data analysis. WC performed testing on the robenidine and EDTA combination, biofilm assay, and data analysis. JS participated in cell cytotoxicity assays. AO contributed to data analysis and manuscript editing. HV and PH contributed to interpretation, analysis, and discussion. AM and SG contributed to discussion, writing and editing. SP conceived the study’s design, and contributed to the writing and editing, and provided financial support for the study. DT contributed to study design, and participated in writing, editing, and discussion, and provided financial support for the study. All authors read and approved the submitted version of the manuscript, in addition to contributing to manuscript revision.

## Conflict of Interest Statement

SP is a director of Neoculi Pty Ltd. DT has received research funding from Neoculi Pty Ltd. The remaining authors declare that the research was conducted in the absence of any commercial or financial relationships that could be construed as a potential conflict of interest.

## References

[B1] Abraham R. J. Stevens A. J. Young K. A. Russell C. Qvist A. Khazandi M. (2016). Robenidine analogues as gram-positive antibacterial agents. *J. Med. Chem.* 59 2126–2138. 10.1021/acs.jmedchem.5b01797 26765953

[B2] Abraham S. Jagoe S. Pang S. Coombs G. W. O’Dea M. Kelly J. (2017). Reverse zoonotic transmission of community-associated MRSA ST1-IV to a dairy cow. *Int. J. Antimicrob. Agents* 50 125–126. 10.1016/j.ijantimicag.2017.05.001 28502696

[B3] Al-Bakri A. G. Othman G. Bustanji Y. (2009). The assessment of the antibacterial and antifungal activities of aspirin, EDTA and aspirin-EDTA combination and their effectiveness as antibiofilm agents. *J. Appl. Microbiol.* 107 280–286. 10.1111/j.1365-2672.2009.04205.x 19302313

[B4] Allam A. Maigre L. Vergalli J. Dumont E. Cinquin B. Alves de Sousa R. (2017). Microspectrofluorimetry to dissect the permeation of ceftazidime in Gram-negative bacteria. *Sci. Rep.* 7:986. 10.1038/s41598-017-00945-8 28428543 PMC5430551

[B5] Arais L. R. Barbosa A. V. Carvalho C. A. Cerqueira A. M. (2016). Antimicrobial resistance, integron carriage, and gyrA and gyrB mutations in *Pseudomonas aeruginosa* isolated from dogs with otitis externa and pyoderma in Brazil. *Vet. Dermatol.* 27 113–117e31. 26833540 10.1111/vde.12290

[B6] Arzanlou M. Chai W. C. Venter H. (2017). Intrinsic, adaptive and acquired antimicrobial resistance in Gram-negative bacteria. *Essays Biochem.* 61 49–59. 10.1042/EBC20160063 28258229

[B7] Bampidis V. Azimonti G. Bastos M. D. L. Christensen H. Dusemund B. Kouba M. (2019). Safety and efficacy of Robenz^®^ 66G (robenidine hydrochloride) for chickens for fattening and turkeys for fattening. *EFSA J.* 17:55. 32626244 10.2903/j.efsa.2019.5613 PMC7009205

[B8] Beck K. M. Waisglass S. E. Dick H. L. Weese J. S. (2012). Prevalence of meticillin-resistant Staphylococcus pseudintermedius (MRSP) from skin and carriage sites of dogs after treatment of their meticillin-resistant or meticillin-sensitive staphylococcal pyoderma. *Vet. Dermatol.* 23 369–375 e66–e67. 22364707 10.1111/j.1365-3164.2012.01035.x

[B9] Bugden D. L. (2013). Identification and antibiotic susceptibility of bacterial isolates from dogs with otitis externa in Australia. *Aust. Vet. J.* 91 43–46. 10.1111/avj.12007 23356371

[B10] Ceri H. Olson M. Morck D. Storey D. Read R. Buret A. (2001). The MBEC assay system: multiple equivalent biofilms for antibiotic and biocide susceptibility testing. *Methods Enzymol.* 337 377–385. 10.1016/S0076-6879(01)37026-X 11398443

[B11] Chambers H. F. Deleo F. R. (2009). Waves of resistance: *Staphylococcus aureus* in the antibiotic era. *Nat. Rev. Microbiol.* 7 629–641. 10.1038/nrmicro2200 19680247 PMC2871281

[B12] Chan W. Y. Hickey E. E. Khazandi M. Page S. W. Trott D. J. Hill P. B. (2018a). In vitro antimicrobial activity of monensin against common clinical isolates associated with canine otitis externa. *Comp. Immunol. Microbiol. Infect. Dis.* 57 34–38. 10.1016/j.cimid.2018.05.001 30017076

[B13] Chan W. Y. Hickey E. E. Khazandi M. Page S. W. Trott D. J. Hill P. B. (2018b). In vitro antimicrobial activity of narasin against common clinical isolates associated with canine otitis externa. *Vet Dermatol.* 29:149-e57. 10.1111/vde.12516 29363210

[B14] Chan W. Y. Khazandi M. Hickey E. E. Page S. W. Trott D. J. Hill P. B. (2018c). In vitro antimicrobial activity of seven adjuvants against common pathogens associated with canine otitis externa. *Vet. Dermatol.* 30:133-e38. 10.1111/vde.12712 30548715

[B15] Clark N. M. Zhanel G. G. Lynch J. P. III (2016). Emergence of antimicrobial resistance among *Acinetobacter* species: a global threat. *Curr. Opin. Crit. Care* 22 491–499. 10.1097/MCC.0000000000000337 27552304

[B16] CLSI (1999). *Methods for Determining Bactericidal Activity of Antimicrobial Agents; Approved Guideline.* Wayne, PA: Clinical and Laboratory Standards Institute.

[B17] CLSI (2015). *Performance Standards for Antimicrobial Disk and Dilution Susceptibility Tests for Bacteria Isolated from Animals* 3rd Edn. Wayne, PA: Clinical and Laboratory Standards Institute.

[B18] Eid S. Y. El-Readi M. Z. Wink M. (2012). Synergism of three-drug combinations of sanguinarine and other plant secondary metabolites with digitonin and doxorubicin in multi-drug resistant cancer cells. *Phytomedicine* 19 1288–1297. 10.1016/j.phymed.2012.08.010 23146422

[B19] Farca A. M. Piromalli G. Maffei F. Re G. (1997). Potentiating effect of EDTA-Tris on the activity of antibiotics against resistant bacteria associated with otitis, dermatitis and cystitis. *J. Small Anim. Pract.* 38 243–245. 10.1111/j.1748-5827.1997.tb03356.x 9200113

[B20] Fernandes M. R. Sellera F. P. Moura Q. Carvalho M. P. N. Rosato P. N. Cerdeira L. (2018). Zooanthroponotic transmission of drug-resistant *Pseudomonas aeruginosa*, Brazil. *Emerg. Infect. Dis.* 24 1160–1162. 10.3201/eid2406.180335 29774849 PMC6004847

[B21] Finnegan S. Percival S. L. (2015). EDTA: an antimicrobial and antibiofilm agent for use in wound care. *Adv. Wound Care* 4 415–421. 10.1089/wound.2014.0577 26155384 PMC4486448

[B22] Gales A. C. Jones R. N. Forward K. R. Linares J. Sader H. S. Verhoef J. (2001). Emerging importance of multidrug-resistant *Acinetobacter* species and *Stenotrophomonas maltophilia* as pathogens in seriously ill patients: geographic patterns, epidemiological features, and trends in the SENTRY antimicrobial surveillance program (1997–1999). *Clin. Infect. Dis.* 32(Suppl. 2) S104–S113. 11320451 10.1086/320183

[B23] Gorla M. C. Cassiolato A. P. Pinhata J. M. W. de Moraes C. Corso A. Gagetti P. (2018). Emergence of resistance to ciprofloxacin in *Neisseria meningitidis* in Brazil. *J. Med. Microbiol.* 67 286–288. 10.1099/jmm.0.000685 29458676

[B24] Guardabassi L. Ghibaudo G. Damborg P. (2010). In vitro antimicrobial activity of a commercial ear antiseptic containing chlorhexidine and Tris-EDTA. *Vet. Dermatol.* 21 282–286. 10.1111/j.1365-3164.2009.00812.x 20030799

[B25] Harrison J. J. Stremick C. A. Turner R. J. Allan N. D. Olson M. E. Ceri H. (2010). Microtiter susceptibility testing of microbes growing on peg lids: a miniaturized biofilm model for high-throughput screening. *Nat. Protoc.* 5 1236–1254. 10.1038/nprot.2010.71 20595953

[B26] Heward E. Cullen M. Hobson J. (2018). Microbiology and antimicrobial susceptibility of otitis externa: a changing pattern of antimicrobial resistance. *J. Laryngol. Otol.* 132 314–317. 10.1017/S0022215118000191 29429416

[B27] Hickey E. E. Wong H. S. Khazandi M. Ogunniyi A. D. Petrovski K. R. Garg S. (2018). Repurposing ionophores as novel antimicrobial agents for the treatment of bovine mastitis caused by gram-positive pathogens. *J. Vet. Pharmacol. Ther.* 41 746–754. 10.1111/jvp.12674 29971788

[B28] Hwang I. S. Hwang J. H. Choi H. Kim K. J. Lee D. G. (2012). Synergistic effects between silver nanoparticles and antibiotics and the mechanisms involved. *J. Med. Microbiol.* 61(Pt 12) 1719–1726. 10.1099/jmm.0.047100-0 22956753

[B29] Kantor S. Kennett R. L. Jr. Waletzky E. Tomcufcik A. S. (1970). 1,3-Bis(p-chlorobenzylideneamino)guanidine hydrochloride (robenzidene): new poultry anticoccidial agent. *Science* 168 373–374. 10.1126/science.168.3929.373 5435895

[B30] Khazandi M. Al-Farha A. A. Coombs G. W. O’Dea M. Pang S. Trott D. J. (2018). Genomic characterization of coagulase-negative staphylococci including methicillin-resistant *Staphylococcus sciuri* causing bovine mastitis. *Vet. Microbiol.* 219 17–22. 10.1016/j.vetmic.2018.04.004 29778192

[B31] Krishnamoorthy G. Leus I. V. Weeks J. W. Wolloscheck D. Rybenkov V. V. Zgurskaya H. I. (2017). Synergy between active efflux and outer membrane diffusion defines rules of antibiotic permeation into gram-negative bacteria. *mBio* 8:e1172-e17. 10.1128/mBio.01172-17 29089426 PMC5666154

[B32] Lozano C. Rezusta A. Ferrer I. Perez-Laguna V. Zarazaga M. Ruiz-Ripa L. (2017). *Staphylococcus pseudintermedius* human infection cases in spain: dog-to-human transmission. *Vector Borne Zoonotic Dis.* 17 268–270. 10.1089/vbz.2016.2048 28075235

[B33] Martin Barrasa J. L. Lupiola Gomez P. Gonzalez Lama Z. Tejedor Junco M. T. (2000). Antibacterial susceptibility patterns of *Pseudomonas* strains isolated from chronic canine otitis externa. *J. Vet. Med. B Infect. Dis. Vet. Public Health* 47 191–196. 10.1046/j.1439-0450.2000.00336.x 10829573

[B34] Mekic S. Matanovic K. Seol B. (2011). Antimicrobial susceptibility of *Pseudomonas aeruginosa* isolates from dogs with otitis externa. *Vet. Rec.* 169:125. 10.1136/vr.d2393 21742683

[B35] Morehead M. S. Scarbrough C. (2018). Emergence of global antibiotic resistance. *Prim Care* 45 467–484. 10.1016/j.pop.2018.05.006 30115335

[B36] Nikaido H. (2003). Molecular basis of bacterial outer membrane permeability revisited. *Microbiol. Mol. Biol. Rev.* 67 593–656. 10.1128/mmbr.67.4.593-656.2003 14665678 PMC309051

[B37] Ofek I. Cohen S. Rahmani R. Kabha K. Tamarkin D. Herzig Y. (1994). Antibacterial synergism of polymyxin B nonapeptide and hydrophobic antibiotics in experimental gram-negative infections in mice. *Antimicrob. Agents Chemother.* 38 374–377. 10.1128/aac.38.2.374 8192470 PMC284461

[B38] Ogunniyi A. D. Khazandi M. Stevens A. J. Sims S. K. Page S. W. Garg S. (2017). Evaluation of robenidine analog NCL195 as a novel broad-spectrum antibacterial agent. *PLoS One* 12:e0183457. 10.1371/journal.pone.0183457 28873428 PMC5584945

[B39] Ozvatan T. Akalin H. Sinirtas M. Ocakoglu G. Yilmaz E. Heper Y. (2016). Nosocomial acinetobacter pneumonia: treatment and prognostic factors in 356 cases. *Respirology* 21 363–369. 10.1111/resp.12698 26635315

[B40] Paluchowska P. Nowak P. Skalkowska M. Bjdak A. (2017). Evaluation of in vitro tigecyclin activity against multidrug-resistant *Acinetobacter baumanni* clinicl isolates from Poland. *Acta Pol. Pharm.* 74 793–800. 29513948

[B41] Paterson S. (2016). Topical ear treatment - options, indications and limitations of current therapy. *J. Small Anim. Pract.* 57 668–678. 10.1111/jsap.12583 27747880

[B42] Pendleton J. N. Gorman S. P. Gilmore B. F. (2013). Clinical relevance of the ESKAPE pathogens. *Expert Rev. Anti. Infect. Ther.* 11 297–308. 10.1586/eri.13.12 23458769

[B43] Rajamuthiah R. Fuchs B. B. Conery A. L. Kim W. Jayamani E. Kwon B. (2015). Repurposing salicylanilide anthelmintic drugs to combat drug resistant *Staphylococcus aureus*. *PLoS One* 10:e0124595. 10.1371/journal.pone.0124595 25897961 PMC4405337

[B44] Rigaut D. Sanquer A. Maynard L. Eeme C. (2011). Efficacy of a topical ear formulation with a pump delivery systemfor the treatment of infectious otitis externa in dogs: a randomized controlled trial. *Int. J. Appl. Res. Vet. Med.* 9 15–28.

[B45] Rougier S. Borell D. Pheulpin S. Woehrle F. Boisrame B. (2005). A comparative study of two antimicrobial/anti-inflammatory formulations in the treatment of canine otitis externa. *Vet. Dermatol.* 16 299–307. 10.1111/j.1365-3164.2005.00465.x 16238809

[B46] Rubin J. Walker R. D. Blickenstaff K. Bodeis-Jones S. Zhao S. (2008). Antimicrobial resistance and genetic characterization of fluoroquinolone resistance of *Pseudomonas aeruginosa* isolated from canine infections. *Vet. Microbiol.* 131 164–172. 10.1016/j.vetmic.2008.02.018 18395369

[B47] Santajit S. Indrawattana N. (2016). Mechanisms of antimicrobial resistance in ESKAPE pathogens. *Biomed. Res. Int.* 2016:2475067. 10.1155/2016/2475067 PMC4871955 27274985

[B48] Saputra S. Jordan D. Worthing K. A. Norris J. M. Wong H. S. Abraham R. (2017). Antimicrobial resistance in coagulase-positive staphylococci isolated from companion animals in Australia: a one year study. *PLoS One* 12:e0176379. 10.1371/journal.pone.0176379 28430811 PMC5400250

[B49] Saravolatz L. D. Stein G. E. Johnson L. B. (2009). Telavancin: a novel lipoglycopeptide. *Clin. Infect. Dis.* 49 1908–1914. 10.1086/648438 19911938

[B50] Schneider E. K. Reyes-Ortega F. Velkov T. Li J. (2017). Antibiotic-non-antibiotic combinations for combating extremely drug-resistant Gram-negative ’superbugs’. *Essays Biochem.* 61 115–125. 10.1042/EBC20160058 28258235

[B51] Selander S. (1969). [Treatment of lead poisoning. Comparison between the effect of sodium calcium EDTA and penicillamine used orally and intravenously]. *Arch. Hig. Rada. Toksikol.* 20:657. 4992048

[B52] Tallarida R. J. (2006). An overview of drug combination analysis with isobolograms. *J. Pharmacol. Exp. Ther.* 319 1–7. 10.1124/jpet.106.104117 16670349

[B53] Tängdén T. Hickman R. A. Forsberg P. Lagerbäck P. Giske C. G. Cars O. (2014). Evaluation of double and triple antibiotic combinations for VIM-and NDM-producing *Klebsiella pneumoniae* by in vitro time-kill experiments. *Antimicrob. Agents Chemother.* 58 1757–1762. 10.1128/AAC.00741-13 24395223 PMC3957864

[B54] Theuretzbacher U. Gottwalt S. Beyer P. Butler M. Czaplewski L. Lienhardt C. (2018). Analysis of the clinical antibacterial and antituberculosis pipeline. *Lancet Infect. Dis.* 19 e40–e50. 10.1016/S1473-3099(18)30513-9 30337260

[B55] van der Kolk J. H. Endimiani A. Graubner C. Gerber V. Perreten V. (2019). *Acinetobacter* in veterinary medicine, with an emphasis on *Acinetobacter baumannii*. *J. Glob. Antimicrob. Resist.* 16 59–71. 10.1016/j.jgar.2018.08.011 30144636

[B56] Vaara M. (1992). Agents that increase the permeability of the outer membrane. *Microbiol. Rev.* 56 395–411. 1406489 10.1128/mr.56.3.395-411.1992 PMC372877

[B57] WHO (2017). *Critically Important Antimicrobials for Human Medicine (5th Revision).* Geneva: World Health Organization.

[B58] Wilcox M. H. (2005). Update on linezolid: the first oxazolidinone antibiotic. *Expert Opin. Pharmacother.* 6 2315–2326. 10.1517/14656566.6.13.2315 16218891

[B59] Worthington R. J. Melander C. (2013). Combination approaches to combat multidrug-resistant bacteria. *Trends Biotechnol.* 31 177–184. 10.1016/j.tibtech.2012.12.006 23333434 PMC3594660

[B60] Zgurskaya H. I. Lopez C. A. Gnanakaran S. (2015). Permeability barrier of gram-negative cell envelopes and approaches to bypass it. *ACS Infect. Dis.* 1 512–522. 10.1021/acsinfecdis.5b00097 26925460 PMC4764994

[B61] Zhanel G. G. Lawson C. D. Zelenitsky S. Findlay B. Schweizer F. Adam H. (2012). Comparison of the next-generation aminoglycoside plazomicin to gentamicin, tobramycin and amikacin. *Expert Rev. Anti. Infect. Ther.* 10 459–473. 10.1586/eri.12.25 22512755

